# Exploring social emotion processing in autism: evaluating the reading the mind in the eyes test using network analysis

**DOI:** 10.1186/s12888-022-03773-x

**Published:** 2022-03-03

**Authors:** Tai-Shan Li, Susan Shur-Fen Gau, Tai-Li Chou

**Affiliations:** 1 Department of Psychology, National Taiwan University, No.1, Sec. 4, Roosevelt Road, Taipei, 106 Taiwan; 2 Department of Psychiatry, National Taiwan University Hospital and College of Medicine, No 7, Chung-Shan South Road, Taipei, 10002 Taiwan

**Keywords:** RMET, Negative emotion, Network analysis, Social cognition

## Abstract

**Background:**

Features of autism spectrum disorder (ASD) include difficulties in processing and interpreting socioemotional information. The "Reading the Mind in the Eyes" test (RMET) is a validated measurement for processing socioemotional ability. However, previous RMET studies did not explore patterns of incorrect answers and the emotional valence of the test items. This study used the Taiwanese version of the RMET and the network analysis methods to examine the differences in underlying mechanisms of socioemotional processes between 30 males with autism spectrum disorder (ASD) (mean age = 18 years) and 30 healthy control males (mean age = 17 years). For each test item, a picture of a person's eyes and partial face was shown with four words describing the emotional status on picture corners. Participants were instructed to choose one of the four words that best matched the person's thinking or feeling. We further classified the words into three valences of emotional categories to examine socioemotional processes.

**Results:**

Our results showed that ASD males performed poorer on the RMET than the controls. ASD males had higher network density and in-degree scores, especially in negative words, than control males.

**Conclusions:**

The findings suggest that males with ASD might have deficits in mapping the best emotional concept words to the target item, especially for processing negative emotion.

## Background

Autism spectrum disorder (ASD) is a heterogeneous group of neurodevelopmental disorders, which present a substantial challenge to explore interrelations across several distinct components of the various severity of symptoms [1, 2]. The core symptoms of ASD included deficits in social communication and social interactions, which are reflected by impairment of social cognition abilities. According to Happe and Frith [3], social cognition can be represented as an elaborate network graph that contains several components, such as emotion processing, empathy, and theory of mind (ToM). Futhermore, Vagnetti et al. [2] found that, compared to typically developing (TD) individuals, the ASD group showed less connection in their constructed network topology of social cognition and had inefficient connections among the components.

Two fundamental components that have often been examined in the social cognition field are cognitive ToM and emotion recognition, also defined as affective ToM [2, 4–6]. Previous studies define cognitive ToM as the ability to infer intentions, dispositions, or beliefs of others and perspectives different from one's own [2, 7]. Affective ToM refers to the individual's ability to identify and discriminate emotional states from the observed signs in the behaviors of others [8–10].

ASD was initially defined as an "affective contact disorder" [11]. This was based on ToM studies that related ASD symptoms with difficulties in processing and interpreting information on socioemotional conditions [12]. There is considerable evidence showing that most of these difficulties for children with ASD could be due to delayed social cognition capacities [2, 3, 13, 14]. Compared to TD children, children with ASD are later to develop these competencies of emerging social cognition capacities in a specific sequence [2, 13, 14].

Deficits in processing social-emotional information have been investigated in numerous ASD studies, showing that individuals with ASD process faces differently from TD individuals [15]. TD infants tend to show preferential attention to more socially revealing features of the face, such as the eyes and the mouth. Increased scanning fixations are directed at the eyes through development [15–17]. In contrast, infants with ASD seem to lack these social predispositions [15, 16]; for instance, less time scanning eyes was found in the case of a 15-month-old infant with ASD [18]. In addition, several eye-tracking studies have shown a deviation in the visual scanning of faces by individuals with ASD [16]. Most significantly, [15] found that individuals with ASD exhibit different viewing strategies while scanning positive and negative facial expressions just like TD individuals. In particular, both groups showed increased scanning of the eye region when looking at faces with negative emotional expressions [15].

The "Reading the Mind in the Eyes" Test (RMET) is one of the most useful measurements of emotion perception, which was used in over 250 studies [2, 7, 19–21]. The RMET was originally developed for the study of high-functioning individuals with autism and their family members [22, 23]. The validity of the RMET is interpreted as measuring a deficit in social cognition between individuals with ASD and TD controls [17, 24], which is also supported by two assumptions: the unique eye information about relevant mental states [19, 25] and the ability to interpret intention and feelings through the eyes [26, 27].

However, few studies have examined the role of words in the RMET. Betz and Barrett [28] compared the forced-choice and free-label versions of RMET, and found that words embedded in the forced-choice of RMET shaped both the semantic and affective interpretation of the RMET eye region stimuli. This finding has implications for the forced-choice version of RMET, suggesting that purported deficits in mental inference in ASD might indicate a deficit in using concepts for mental states. Therefore, all the words embedded in the forced-choice RMET could be linked to specific concepts for mental states, which leads all the choice behavior represented the observer's mental states about the observed emotions and worth to be explored. Comparisons of accuracy or total scores for the RMET between groups in previous studies could only reflect the lower mental inference capability of the poor performance group, which leaves the inner process of mental inference unknown. Thus it is crucial to explore the meaning of error words and stimuli matching behavior through the RMET.

In contrast to previous RMET studies focusing on correct answer analysis, the underlying mechanisms of error answers are first explored using complex network approaches [29]. The complex network analysis is based on the constructed graph, which contains well-defined nodes (answers for each item) and edges (participants' choices). Graph theory explores the associations among interacting entities in the complex network. For example, social cognition can be understood as a complex network diagram. The graph theory approach of social cognition includes several distinct but interrelated components, such as emotion processing, empathy, and ToM, etc. Therefore, as in the RMET test, social-emotional information processing cannot be considered a single and independent process but rather as a complex construct in which the different components working together is still unclear [2].

Consequently, to better understand the differences in social emotion processing between male participants with autism typically developing, a complex construct network analysis approach is utilized in the present study. Using emotion words in the RMET as nodes, and the answering behavior as edges, enables us to firstly explore emotional facial expressions and emotional word mapping processes in male participants with autism and in typically developing controls. We also firstly investigate the difference in patterns between male participants with autism versus typically developing controls when processing the various valence of emotional expressions.

## Methods

### Participants

The present study comprised 30 males with autism spectrum disorder (ASD) (mean age = 18 years) and 30 healthy male controls (mean age = 17 years). Male participants with autism, recruited from the Department of Psychiatry, National Taiwan University Hospital, were diagnosed by the corresponding author, a senior child psychiatrist, before recruitment, according to the DSM-IV and ICD-10 diagnostic criteria. The male participants with autism were matched to controls by age, sex, and IQ [30]. Clinical diagnosis of male participants with autism was further confirmed by interview using the Chinese version of the Autism Diagnostic Interview-Revised (ADI-R) [31–33]. Both groups of participants were also assessed by the Chinese version of the Autism Spectrum Quotient (Chinese AQ) [34] and interviewed to ensure that they had no history of other psychiatric disorders. All participants that we recruited were native Mandarin-Chinese speakers with normal hearing, and normal or corrected-to-normal vision. The Research Ethics Committee at the National Taiwan University Hospital approved this study (Approval number, 201403109RINC; ClinicalTrials.gov number, NCT02233348). Written informed consent was obtained from all the participants and their parents (Table 1).

**Table 1:** Demographic information and performance of the RMET

**Characteristics**	**ASD (*n* = 30)**	**Controls (*n* = 30)**	**Significant test**	***P* value**
**Demographic**				
**Age (mean years ± S.D.)**	17 ± 4	18 ± 5	*t*(58) = -1.29	0.18
**VIQ (V)**	101 ± 36	107 ± 23	*t*(58) = -0.84	0.40
**PIQ (P)**	112 ± 12	108 ± 22	*t*(58) = -0.69	0.56
**FIQ (F)**	100 ± 35	109 ± 23	*t*(58) = -1.15	0.20
**The RMET performance**				
**Accuracy (mean ± S.D.)**	68 ± 11	74 ± 8	*F*(1,58) = 5.03	0.03
**Reaction time (mean ± S.D.)**	4006 ± 438	3485 ± 671	*F*(1,58) = 23.25	0.00

*Note: VIQ* Verbal Intelligence, *PIQ* Performance Intelligence, *FIQ* Full Intelligence

### Behavioral measure assessments and analyses

**Fig. 1:**
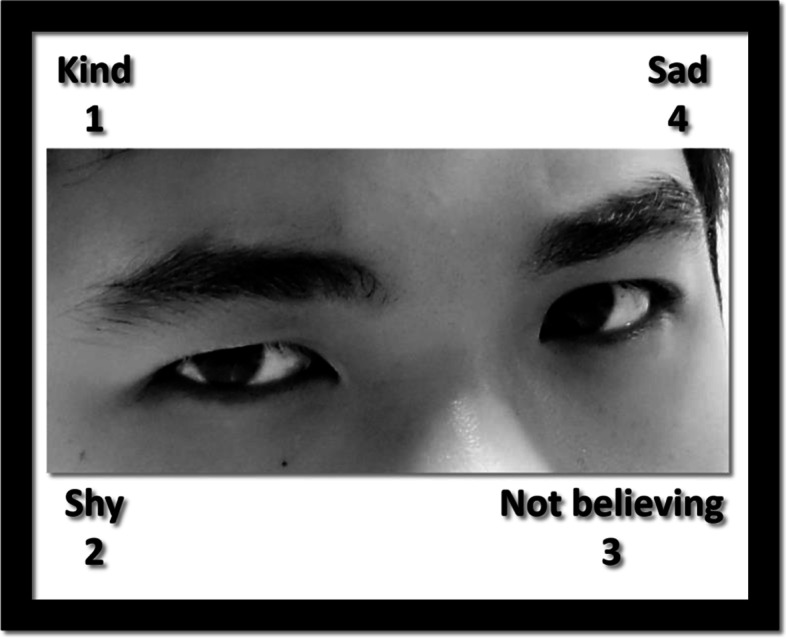
An example of stimuli in the Eyes task. Four words of descriptions were randomly located in the corners. Participants were instructed to choose which word best matched what the person was thinking or feeling. Choice 1: kind; choice 2: shy; choice 3: not believing, and choice 4: sad. The correct is the choice 3. (Consent from the individual for publication of this image was obtained)

The Taiwanese version of the RMET [29] was presented on a laptop computer. Each participant took approximately 6 min to complete five practice items and 43 test items. For each item, a picture of a person's eyes and partial face was shown with four words on picture corners for 8 s. Participants were instructed to choose one of the four words best matching with the person's thinking or feeling [35]. Thus, the RMET was based on a four-alternative forced-choice paradigm, with a 25% correct guess rate. An example of the trials is shown in Fig. 1. All the pictures were taken from six professional actors/actresses in Taiwan performing various mental states. Their eye regions were standardized to a similar size and gray-scale. For words on picture corners, the target words [i.e., the target words of item number 20 is "despondent"] to describe the mental conditions of pictures were based on the original response items (combined with the adult and child versions) [19, 25].

Accuracy was computed by summing up the frequency of correct responses from 43 items, and reaction time was measured from the onset of each item. One-way ANOVA was used in the present study to examine differences between two groups (male participants with autism and typically developing controls) regarding accuracy and reaction time measures. To further explore the relationship between the Taiwanese version of the RMET and autistic traits, we used Pearson's correlation to evaluate the association between behavioral measures from the RMET and the Chinese version ADI-R scores in the male participants with autism group. We also analyzed the association between performance on the Taiwanese version of the RMET and scores on the Chinese version AQ in both groups.

### Network measure analysis

To further explore mental states' internal structures, we used full complexity analysis to construct the network of choice patterns, including two types of building blocks: nodes and edges. Nodes, usually visualized as circles, represent any conceivable variable (e.g., symptoms, persons, airports, neurons). Edges, the linkages between these nodes, represent any conceivable relationships [36]. To build this network, we first identified the words that would function as nodes. Then we determined the relationship of participants' choices (i.e., the correct answer and wrong answers) to be represented by the edges. Any two words of participants' choices were connected in the network. These connections were coded in a binary matrix and subsequently used as an input with the Gephi version 0.9.2 (Gephi.org) software for visualizing the network [37].

The correct answer for each item functioned as a target node, while all four candidate words and no choice condition of the item were treated as source nodes. Connections between the target node and source nodes were directional, indicating the direction from source to target. Based on this directional influence, two explanatory indicators (density, in-degree) of the network were calculated [29].

Density was defined as the actual connections divided by potential connections (all possible connections) in a network. There were 5 possible answers (i.e., 5 nodes) and 10 potential connections for a given item. If no actual connection existed, the density would be zero, and if actual connections were the same as potential connections, the density would be 1. Thus, density ranged between 0 and 1. A lower density score indicated fewer connections (i.e., a more consistent match between a word and the picture), and a higher density score indicated more diverse answers (i.e., less consensus to the picture) across all participants.

In-degree: In-degree was an item-based measure that counted connections from all nodes (all possible answers) to a particular node (the correct answer). In the present study, an in-degree score ranged from 1 to 5 for a given item, and there were 43 items. There were 5 different answers for a given item, including no response, the correct answer, and 3 wrong answers across all participants. For example, score 1 indicated only an answer to be chosen by all participants, while score 5 indicated all possible answers to be chosen by all participants. Therefore, in-degree scores between 1 and 5 indicated the diversity of answers inferring the mental state of a picture, the score 1 for an entirely consistent match between a word and the picture, and the score 5 for the least consensus across all participants. Because the 43 items in the RMET were more than 30, we conducted a two-sample t-test (parametric statistics) to compare the in-degree difference between the two groups.

### Emotional word classification

To explore whether there were different properties of emotion polarity between two groups of male participants, we divided the 43 target words into three-item groups based on emotion polarity. According to the Chinese sentimental database NTUSD [38], 44% (*n* = 19) target words were tagged as negative words, 21% (*n* = 9) were tagged as positive words, and 35% (*n* = 15) were tagged as neutral words. The selected stimuli were rated using a questionnaire with 106 healthy adult participants (mean age = 25 years, SD = 5, females = 49%) in Taiwan. In the questionnaire, participants were asked to judge the association between pairs of a picture and a target word, ranging from 1 (not associated) to 5 (strongly associated). The inter-rater reliability was measured across items within each category. Cronbach's alpha measured the internal consistency of three categories using SPSS-PC version 19 (SPSS, Inc) software for the rating scores of 106 healthy adult participants. The Cronbach's alpha was 0.77 for negative items, 0.75 for neutral items, and 0.60 for positive items [29]. The mean score across 106 participants was 3.64 across categories, and the standard deviation (SD) was 1.04. For negative items, the mean score was 3.53 (SD = 0.32); for neutral items, the mean score was 3.71 (SD = 0.72); and for positive items, the mean score was 3.77 (SD = 0.57). We used an independent *t*-test to determine a statistically significant difference between the ASD and control groups.

## Results

### Behavioral performance

Demographic characteristics for male participants with autism and typically developing controls are presented in Table 1. A one-way ANOVA for accuracy was significant between the ASD and control groups, [*F*(1, 58) = 5.03, *p* < 0.01], with the male participants with autism group being less accurate than the male participants with typically developing controls. Moreover, a one-way ANOVA for reaction time was significant [*F*(1, 58) = 23.25, *p* < 0.001], with the male participants with autism group being slower than the typically developing controls group.

Correlation between the accuracy of the RMET and the Patterns subscale of AQ showed a negative relationship (*r* = -0.441, *p* < 0.05) in the male participants with autism group. A negative correlation was also found between the accuracy of the RMET and socio-emotional reciprocity of the ADIR (*r* = -0.427, *p* < 0.05) in the ASD group.

### Network analysis

The total number of the forced-choices performed by a participant was recorded to create a binary matrix for each item. Each value in the binary matrix portrayed the raw choices made by the participants. The response distribution network was constructed from this matrix and presented for the male participants with autism group and the typically developing controls group separately.

**Fig. 2:**
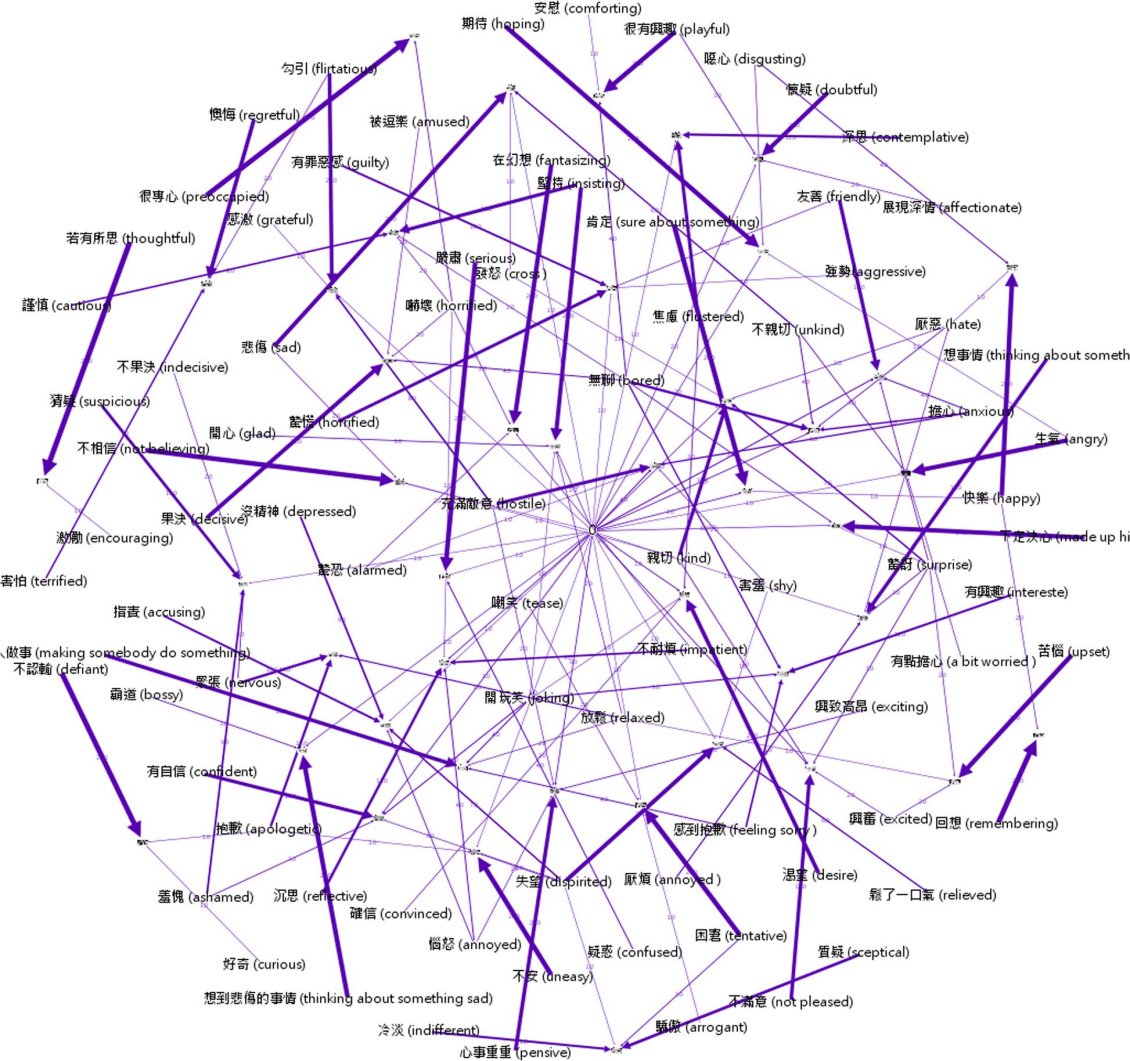
Response distribution network of the ASD group. Photographical nodes in the network represent the 43 target words; word-form nodes in the network represent the source words; edges between these nodes represent the relationship of participants' choices between target words and source words: The more times that a word was chosen by participants, the thicker the edge. Density is the actual connections divided by potential connections (all possible connections) in network. In the present study, there were 5 possible answers (i.e., 5 nodes) and 10 potential connections for a given item. If no actual connection exits, density is zero; In-degree is an item-based measure that counts connections from all nodes (all possible answers) to a particular node (the correct answer)

**Fig. 3:**
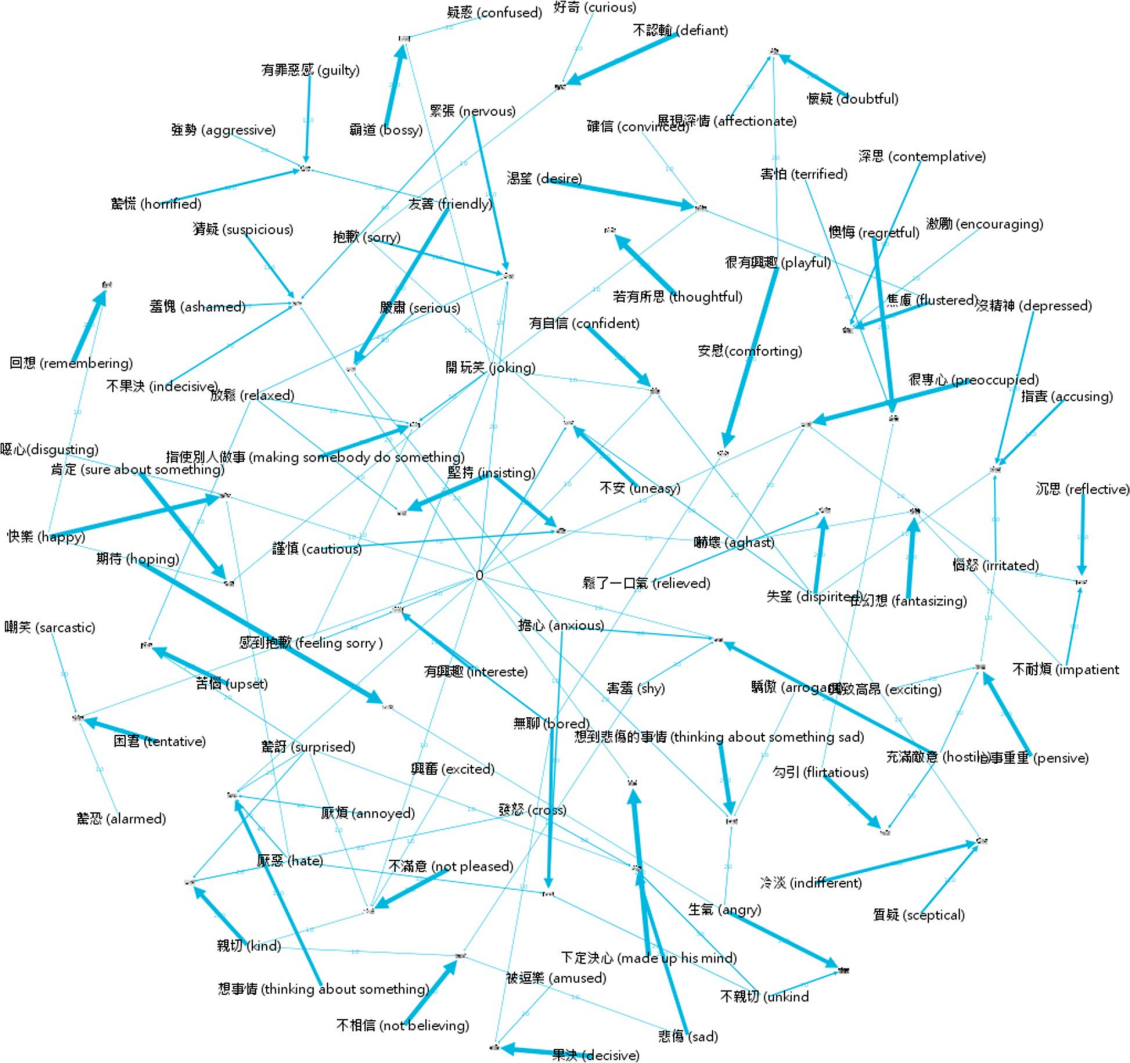
Response distribution network of the control group. The photographical nodes in the network represent the 43 target words; word-form nodes in the network represent the source words; edges between these nodes represent the relationship of participants' choices between target words and source words: The more times that a word is chosen by participants, the thicker the edge. Density is the actual connections divided by all possible connections in the network. In the present study, there were 5 possible answers (i.e., 5 nodes) and 10 potential connections for a given item. If no actual connection exits, density is be zero; In-degree is an item-based measure that counts connections from all nodes (all possible answers) to a particular node (the correct answer)

Density: The response distribution network of the male participants with autism group (Fig. 2) and the male participants with typically developing controls group (Fig. 3) shows participants' forced-choices and the correct answers (target words) of each item as connected nodes. The two group networks were analyzed for structure diameter density, and Table 2 indicates the network diameter values of these two networks. Compared with controls, the male participants with autism showed greater density in the network structure, which was considered more diversity in their choice patterns.

**Table 2:** In-degree and emotion polarity for controls and ASD participants

**Target term**	**ASD**	**Controls**	**Significant test**	***P* value**
**Number of nodes**	127	124	-	-
**Number of edges**	170	146	-	-
**Network density**	0.011	0.010		
**In-degree (mean score ± S.D.) (*n* = 43)**	4 ± 1	3 ± 1	*t*(84) = 2.75	0.01
**Polar**				
**Negative (mean score ± S.D.) (*n* = 19)**	4 ± 0	3 ± 1	*t*(36) = 2.46	0.02
**Neutral (mean score ± S.D.) (*n* = 15)**	3 ± 1	3 ± 0	*t*(28) = 1.10	0.28
**Positive (mean score ± S.D.) (*n* = 9)**	4 ± 0	3 ± 1	*t*(16) = 1.41	0.18

*Note:* Nodes: words (i.e., all the words appearing in the experiment, including the 43 target words and all answers);. Edges: relationship of participants' choices (i.e., the correct answer and wrong answers); Density: defined as the actual connections divided by all possible connections in network. In the present study, there were 5 possible answers (i.e., 5 nodes) and potential connections would be 10 for a given item. If no actual connection exits, density is zero; In-degree: an item-based measure that counts connections from all nodes (all possible answers) to a particular node (the correct answer)

In-degree: In the response distribution network, in-degree measures provided information about the number of a word that participants chose as the correct answer to match the mental state of a picture. In this measure, the in-degree scores indicated the two groups' differential response distribution (Fig. 2 and Fig. 3). There was a significant difference for the in-degree scores between these two groups, [*t*(84) = 2.75, *p* < 0.01], with the male participants with autism group having higher scores than the ypically developing controls group, indicating that the male participants with autism group had less centrality (i.e., more diversity) than ypically developing controls.

The network edges (Table 2) show that the male participants with autism group contained more edge numbers than the typically developing controls group, indicating more wrong answers for the male participants with autism group.

### Emotional word classification

Compared to the control group, the male participants with autism group showed more diverse choices of negative words. The in-degree scores of negative words were significantly different between these two groups, [*t*(36) = 2.46, *p* < 0.05], with the male participants with autism group having more different choices than the typically developing controls group. However, there was no significant difference between these two groups for the in-degree scores of neutral words [*t*(28) = 1.10, *p* = 0.28] or positive words [*t*(16) = 1.41, *p* = 0.18].

## Discussion

This study compared social emotion processing between male participants with autism and typically developing controls using the Taiwanese version of the RMET. In contrast to previous studies, where there was less emphasis on analyzing emotional words in the RMET, our present study highly valued the role of emotional words. Based on the emotional expression of eyes region stimuli and mapping processing for emotional words, forced-choice performances were analyzed and compared across male participants with autism and typically developing controls groups. In order to observe the mapping processing of two groups' forced-choice patterns, we first utilized complex network analysis and two network measure indexes to take wrong answers into account. Indeed, all the forced-choice performances included wrong answering behavior, defined as a mapping process representing the mental concepts of emotion recognition.

The present study provides the clinical evidence of mapping emotional words to eye expressions. The main contribution lies in providing stronger evidence of strengthening the link between ToM and emotion recognition, which was relatively under-investigated in prior studies [17]. Recently, Altschuler et al. [39], found the distinct association between face perception and affective ToM in male participants with autism that may not appear in male participants with typically developing controls. Our results further suggest distinct relations across three valences of emotional categories.Therefore, this study provides new clinical evidence for different emotional word mapping strategies in male participants with autism while mapping the words to the negative emotional expressions of eyes.

Network analysis enables exploration of the internal structures of mental inference processing. For this analysis, two measure indexes, network density and in-degree score, were used to examine the characteristics of the participants' answering patterns. In the present study, network density represents the participants' answering diversity. The other index, in-degree, is an item-based measurement, which provides quantitative value for comparing answering patterns of two groups. We found that network density and in-degree scores of male participants with autism were both higher than typically developing controls, consistent with less accurate and longer reaction time in male participants with autism. These findings support the inference on the difficulty of processing emotional expressions in male participants with autism [17, 40].

Our main focus is on the effect of different valences of emotional expressions on answering patterns. We classified the 43 target words into positive, neutral, and negative valences to further explore these associations between answering patterns and different types of emotion. Our results showed that in-degree scores were significantly different between the two groups in negative emotion target words rather than neutral and positive ones. Our findings generally support the emotion-specific deficits observed in the previous studies. Male participants with autism were found to perform slower in recognizing emotion and to selectively make more errors in detecting negative emotions from facial expressions, such as anger, fear, sadness, and disgust [41–45]. On the other hand, they were found less effectively than male participants with typically developing controls while processing the information from the face's eye region when attempting to recognize emotions [22, 46].

In the RMET, participants' responses for recognizing emotions could indicate both scanning and mapping strategies for processing pictures and words. For scanning strategies, although the clinical definitions and empirical evidence proposed "Eye Avoidance" hypothesis of ASD face processing [47, 48], several studies still point to the extended scanning of the eye region preference of them [49, 50]. For instance, [15] provided evidence of similar viewing strategies exhibited in TD individuals and individuals with ASD when scanning negative facial expressions. Both groups showed increased scanning of the eye region when looking at faces with negative emotional expressions. Mapping strategies in the RMET are related to cross-modal integration and requiring verbal labeling of emotional eyes ability, which was shown to be challenging for individuals with ASD [44, 51, 52]. Unlike the above studies that reported such challenge might be mainly due to immature verbal ability, our results provide another observation by well controlling the verbal IQ scores between male participants with autism and typically developing controls. We found that male participants with autism showed a deficit only in mapping verbal labels to negative emotional eyes but performed similarly to typically developing controls with neutral and positive emotional eyes (also see [40, 53, 54]. Our finding of the poor RMET performance in male participants with autism suggested that male participants with autism failed to map the observed emotional eyes onto the representations of those emotional words. Such mapping process is essential evidence for representing what the observer actually feels about the observed emotions [15]. Thus, ASD males may have deficits in mapping rather than scanning negative emotion expression.

Accordingly, negative emotions result from appraisals of goal incongruence and might trigger more detail-oriented processing [55, 56]. Based on the feeling-as-information theory of emotion [57, 58], negative emotion triggers a distinct, bottom-up, and systematic processing in which an individual engages in a more fine-grained, detailed analysis of experience. Positive emotion, on the other hand, triggers a congruent, top-down, and heuristic processing in which an individual relies on the more general knowledge structures [56]. In the present study, we found this valence-specific impairment in negative emotion recognition in male participants with autism, which is consistent with some previous studies that have reported deficits in recognizing negative facial emotions [41, 59]. Yeung et al., [59] found that children with ASD might be able to gradually develop skills in recognizing positive rather than negative emotions. That is, positive emotions might be easier to identify and more congruent than negative emotions. The age-related results of Yeung et al., [59] might partly explain the impairment in recognizing negative but not positive facial expressions in adolescents with ASD.

In addition to the age-related explanation, another interpretation of these differential results is that participants with ASD might have failed to map the observed emotions onto representations of those emotions [15]. Accordingly, words play an important role in shaping mental inferences by the special type of sensory input that is inextricably linked to concepts and categories [28]. The systematic review of previous research on emotional language processing in ASD by Lartseva et al., [60] summarized that individuals with ASD can correctly identify words as emotionally positive or negative, but have difficulty providing an in-depth explanation of them.

Taking item number 20 as an example, we found the eye region stimuli present a negative emotional expression, which matches the target answer of the emotional word "despondent". Most participants in the control group (*n* = 24, 70%) correctly chose the emotional word "despondent", and only 6 controls chose the other emotional word "relief" to match the despondent eyes expression picture. Compared to the male participants with typically developing controls group, there is more diversity in patterns chosen by the male participants with autism group. Male participants with autism not only chose the emotional words "despondent" and "relief " but also "shy" or even failed to choose a word to match the target stimuli. Accordingly, the linguistic ambiguity of negative emotional words might increase the difficulty of forced-choice tests, such as the RMET used in the present study. Moreover, impoverished emotional concepts and limited emotional vocabularies are also related to the purported mental inference deficits in participants with ASD [28].

The RMET is a widely used measure of affective ToM across clinical and nonclinical populations, and its validity is supported by strong associations with clinical changes in psychosis as well as the severity of ASD social symptoms [61, 62]. In the assessment of concurrent validity of this study, we also examined the association between these behavioral measures and autistic traits using the Chinese versions of ADI-R and AQ. The results reveal the association between socio-emotional reciprocity impairment and performance in the RMET by male participants with autism. That these participants have greater socio-emotional reciprocity impairment is correlated to their less accurate performance on the RMET. According to the defined criteria of the social reciprocity domain of the ADI-R, socio-emotional reciprocity includes the use of the body to communicate, offering comfort, quality of expression of social interest, appropriate facial expressions, and social response [63].

In agreement with the present studyThe, Baron-Cohen, Wheelwright [64], and Voraeck et al. [65] reported a significant negative correlation between the AQ scores and the accuracy performance in the RMET. In particular, we found one AQ subscale (Pattern) was negatively related to accuracy in the male participants with autism group, indicating that higher restricted interests behavior is correlated to less accurate performance on the RMET by male participants with autism. These findings suggest that the lack of ability to recognize emotional facial expressions might be related to the lack of socio-emotional reciprocity ability and restricted interests behavior patterns in male participants with autism.

In summary, using the Taiwanese version of the RMET, the present study demonstrated that male participants with autism were significantly more impaired than typically developing controls at correctly identifying emotional states in others. Notably, we constructed a complex network of two groups for both qualitative explanation and quantitative comparison. We also considered the emotional valence in emotion processing tests, which provided the first evidence for different emotion processing patterns in male participants with autism when mapping negative emotional expressions. Using the in-degree index, we directly found that male participants with autism had higher in-degree scores than male participants with typically developing controls, especially for eyes expressing negative emotions, presenting a more diverse choice pattern in the male participants with autism group for matching negative emotion words.

However, the in-degree scores can only show whole group performances without individual differences. This poses a potential limitation since in-degree scores might have varied from only few participants' choices. For instance, in each item, if five participants in the groups make five different choices, the in-degree scores will be five, no matter how many participants actually cause these differences. The in-degree index is limited by the lack of variation information on the individual choice patterns in the group. Future studies could attempt to consider the weight calculation of each edge in the network to provide more understanding of the group differences.

The other limitation of the present study is a relatively wide age range with a single gender, which may restrict the ability to explore more variables, such as gender, development difference or severity of symptoms. Our results should be interpreted with caution. A future study with a age group separation would be needed. Additionally, male participants with autism although our version has more items (*N* = 43) than other versions (*N* = 22 ~ 36), we do not get an equal proportion of emotion polarity for negative, neutral, and positive items. A future study is needed to address this issue.

Male participants with autism present a heterogeneous group of disorders, characterized by impaired social cognition. This presents a substantial challenge to explore the interrelation across several distinct components that refers to the capacity to process information about social emotional context. Since sensitivity to emotional expressions emerges very early in development [66, 67], social brain involvement is thought to be the key component in early ToM development [41, 53]. To determine the characteristics of emotion recognition ability in ASD, future studies should examined the activation of social brain areas while doing a ToM related task. Emotion recognition impairments in ASD might be linked with impairments in early ToM development.

## Data Availability

The datasets used and analyzed during the current study are available from the corresponding authors (clnical data with SSG, eyes data with TLC) on reasonable request.

## Abbreviations

RMET: Reading the Mind in the Eyes.
ASD: Autism spectrum disorder.
TD: Typically developing
